# Insights into the H_2_/CH_4_ Separation Through Two-Dimensional Graphene Channels: Influence of Edge Functionalization

**DOI:** 10.1186/s11671-015-1199-2

**Published:** 2015-12-23

**Authors:** Jing Xu, Pengpeng Sang, Wei Xing, Zemin Shi, Lianming Zhao, Wenyue Guo, Zifeng Yan

**Affiliations:** College of Science, China University of Petroleum, Qingdao, Shandong 266580 People’s Republic of China; State Key Laboratory of Heavy Oil Processing, Key Laboratory of Catalysis, China University of Petroleum, Qingdao, 266580 People’s Republic of China

**Keywords:** Molecular dynamics, Gas separation, Graphene membrane, Two-dimensional channel

## Abstract

A molecular simulation technique is employed to investigate the transport of H_2_/CH_4_ mixture through the two-dimensional (2D) channel between adjacent graphene layers. Pristine graphene membrane (GM) with pore width of 0.515~0.6 nm is found to only allow H_2_ molecules to enter rather than CH_4_, forming a molecular sieve. At pore widths of 0.64~1.366 nm, both H_2_ and CH_4_ molecules could fill into the GM channel, where the permeability of methane is more preferential than that of hydrogen with the largest CH_4_/H_2_ selectivity (1.89) at 0.728 nm. The edge functionalization by –H, –F, –OH, –NH_2_, and –COOH groups could significantly alter gas permeability by modifying the active surface area of the pore and tuning attractive and/or repulsive interaction with molecules at the entrance of channel. At the pore width of 0.6 nm, the H_2_ permeability of molecular sieve is enhanced by –H, –F, and –OH groups but restrained by –NH_2_, especially –COOH with a passing rate of zero. At pore widths of 0.64 and 0.728 nm, both –H and –F edge-functionalized GMs show a preferential selectivity of methane over hydrogen, while the favorable transport for GM–OH is changed from H_2_ molecules at 0.64 nm to CH_4_ molecules at 0.728 nm. For GM–NH_2_, it exhibits an excellent hydrogen molecular sieve at 0.64 nm and then turns into a significant H_2_/CH_4_ selectivity at 0.728 nm. Meanwhile, small H_2_ molecules start to enter the channel of GM–COOH at the pore width up to 0.728 nm. For the largest pore width of 1.336 nm, the influence of edge functionalization becomes small, and a comparable CH_4_/H_2_ selectivity is observed for all the considered membranes.

**Electronic supplementary material:**

The online version of this article (doi:10.1186/s11671-015-1199-2) contains supplementary material, which is available to authorized users.

## Background

In the past few decades, membrane separation technologies exhibit many fascinating properties including low energy consumption, facile operation, and high cost effectiveness and thus have attracted much research attention [1–3]. Among various membrane materials, graphene-based materials have the two-dimensional (2D) carbon sheets with large surface area, chemical stability, mechanical robustness, and high impermeability, and thus, they are considered as one of the most potential classes of separation membranes [4–11]. Because of the high impermeability, the perfect graphene is a good barrier layer for gases and liquids. For molecular separation, therefore, a graphene-based membrane needs to be functionalized with nanopores or nanochannels.

For the porous membranes, the selective molecular permeation could be enabled by opening and controlling the holes on the 2D graphene sheet. In the real-word, however, it is extremely difficult to fabricate a large-area monolayer graphene material with controllable and uniform high-density nanopores. Alternatively, we could prepare a separation membrane with stacked 2D graphene sheets, and the gas molecules could selectively permeate through the 2D channels by controlling the interlayer spacing. Now, the interlayer channel size could be tuned by oxidation [4] and intercalating different-sized cross-linking molecules [12, 13], nanoparticles [14], and nanowires [15].

The 2D channels between two stacked graphene nanosheets may allow the special molecules to pass through but reject the unwanted molecules. For example, Qiu et al. reported that the nanochannels within chemically converted graphene sheets can be controlled by hydrothermal treatment, leading to the selective passing of water and small metal nanoparticles [16]. Nair et al. found that the submicrometer-thick graphene oxide membranes could completely reject liquids, vapors, and gases (He, Ar, H_2_, and N_2_), but water is allowed to permeate facilely [17]. They suggested that this seemingly incompatible phenomenon is attributed to a low-friction flow of a monolayer of water through two-dimensional capillaries formed by closely spaced graphene sheets [17]. Recently, Li prepared an ultrathin 1.8-nm-thick graphene oxide membrane by a facile filtration method, which exhibits the ultra-high selectivity for H_2_/CO_2_ and H_2_/N_2_ mixtures [18]. In addition, the permeability and selectivity of graphene-based membranes to ions and molecules have also been investigated in aqueous media [18].

To explore the mechanism of molecular transport, molecular dynamic (MD) simulation, as a powerful tool, has been employed. For 2D graphene channels, MD simulation showed that the water molecules cannot fill the 2D graphene capillaries with interplanar distance below 0.6 nm, whereas the capillaries with interplanar distance ranging from 0.6 to 1.0 nm could be filled by one and two layers of water molecules [17]. Vieira-Linhares and Seaton studied the transport mechanism of H_2_/CH_4_ mixture in the 2D graphite channels by a combination of grand canonical molecular dynamics (GCMC) and dual control volume GCMC (DCV-GCMD) methods [19]. They found the 2D channels show a sieving effect below 0.6 nm, significant selective adsorption of methane between about 0.63 and 1 nm, and poorer separation bigger than 1.0–1.2 nm [19]. Furukawa and Nitta investigated that the permeation of pure and mixed gases (CH_4_ and C_2_H_6_) across carbon membranes with the pore shapes of diamond, zigzag, and straight paths by the nonequilibrium molecular dynamics simulations (NEMD) [20]. Xu et al. performed NEMD simulations on the effect of temperature on the transport and separation of the CO_2_/CH_4_ gases through a 2D carbon nanopore [21]. By the NEMD technique, MacElroy and Boyle studied the effect of pressures on the transport of binary H_2_/CH_4_ mixtures through a model slit carbon membrane [22]. Recently, Jin et al. reported the flow of methane in the nanochannels of graphite layers at low and high pressures by DCV-GCMD simulations [23].

Theoretical investigations suggested that the nature of the functional groups at the edges of the pores plays a key role in the selective molecular separation for the porous single-layer graphene [6, 24, 25]. Nevertheless, to our best knowledge, the investigation of the influence of the functionalization at the sheet edge on the molecular permeation and separation is rather scarce for the 2D graphene channels. In this work, a molecular simulation technology is used to systematically study the effect of edge functionalization on the H_2_/CH_4_ separation through 2D graphene channels with width of 0.515~1.366 nm.

## Methods

**Fig. 1:**
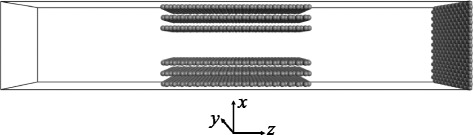
Model of the simulation box. The origin is located at the center of the box

Molecular models of pristine graphene membrane (GM) and GM with edge modified by functional –H, −F, −OH, −NH_2_, and –COOH groups were established using Materials Studio [26]. The GM modelled in this work is shown in Fig. 1. The simulation box consists of three basal graphite layers above and below a single isolated pore. Periodic boundary conditions were applied in three dimensions. Therefore, there are six graphite layers between two pores in the *x* direction, while the membrane is of infinite breadth in the *y* direction. The length of channel in the *z* direction is 4.92 nm for pristine GM. The size of simulation box is (1.7 + W) nm × 4.26 nm × 15.028 nm in the *x*, *y*, and *z* directions, respectively, where *W* is the pore widths of 0.515, 0.6, 0.64, 0.728, 0.855, 1.111, and 1.366 nm. The GM was placed in the middle of the box, and the gas phase and vacuum phase were divided by the fixed GM. The gas phase was the mixture of H_2_ and CH_4_ with 1:1 composition and density of 0.03269 g cm^−3^ at the initial step, similar to the values reported by Tao et al. [27].

MD calculations were performed using the Discover code in Materials Studio of Accelrys Inc. [26]. The interatomic interactions are described by the force field of a condensed-phase optimized molecular potential for atomistic simulation studies (COMPASS) [28]. COMPASS is a first ab initio force field, which has presented a good reliability in describing the absorbate–adsorbent interaction for the gas molecules in carbon-based materials [24, 27]. The cutoff distance for truncation of the intermolecular interactions was set to 1.28 nm, and the Ewald sum technique was used to calculate the electrostatic interaction. The Andersen thermostat method was used to control the temperature of the system at 300 K [29]. MD simulations were carried out in the canonical (NVT) ensemble. During the simulations, the time steps were set to 1 × 10^7^, with a fixed time step of 1 fs. Data was collected every 5 ps.

Electron density was calculated using PBE functional with the double-ξ numerical polarization (DNP) basis set, which was implemented in the DMol^3^ code in the Materials Studio of Accelrys Inc. [30, 31]. The tolerances of energy, gradient, and displacement convergence were 1 × 10^−5^ hartree, 2 × 10^−2^ hartree/nm, and 5 × 10^−4^ nm, respectively.

The selectivity of component *i* over component *j* (*S*_*i*/*j*_) is defined by the following schematic equation:

$$ {S}_{i/j}=\left({x}_i\;/\;{x}_j\right)\;/\;\left({y}_i\;/\;{y}_j\right) $$

where *x*_*i*_ (*x*_*j*_) is the mole fraction of component *i* (*j*) entering the pore and *y*_*i*_ (*y*_*j*_) are the mole fractions of component *i* (*j*) in the gas phase.

## Results and Discussion

### Pristine Graphene Membranes

#### W = 0.515 − 0.6 nm

**Fig. 2:**
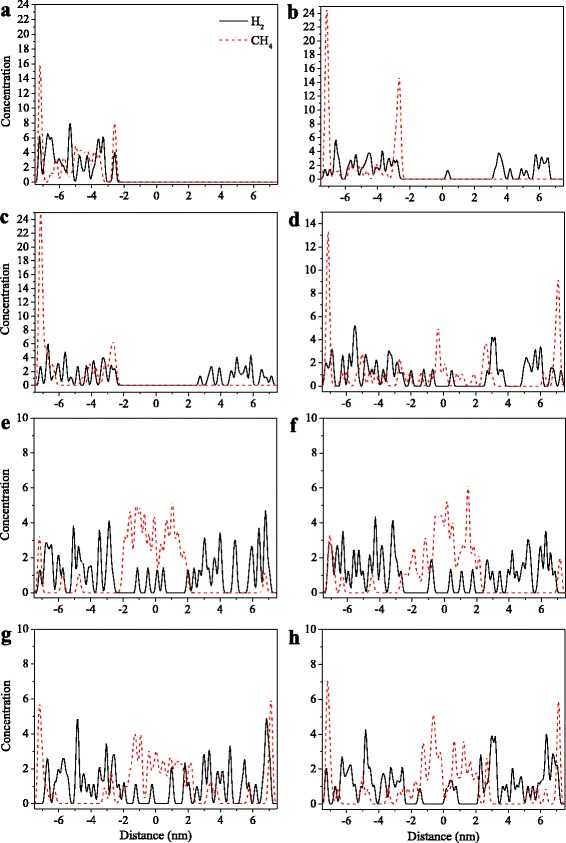
Concentration profiles of the 1:1 H_2_/CH_4_ mixture permeating pristine GM along the *z* direction for **a** the initial configuration at pore width of 0.515 nm and the final configurations at pore widths of **b** 0.515, **c** 0.6, **d** 0.64, **e** 0.728, **f** 0.855, **g** 1.11, and **h** 1.366 nm

The amount of gas molecules entering the channel of GM is shown in Table 1. The final configurations of the gas molecules permeating GMs are given in Additional file 1: Figure S1. Because of a small kinetic diameter of H_2_ molecule (0.283 nm), 55 molecules are allowed to enter the 2D channel of 0.515 nm at 10 ns (see Table 1). When the pore size increases to 0.6 nm, more H_2_ molecules (61) can diffuse into the channel. At pore sizes of 0.515 and 0.6 nm, the concentration profile indicates that the stray H2 molecules are distributed throughout two boxes without obvious accumulation in the channel or near the edges of the membrane (Fig. 2b, c), suggesting most of H_2_ molecules could pass the membrane to the side vacuum boxes with trace amount of adsorbed species in the channel. For CH_4_ molecules (kinetic diameter of 0.376 nm), they are found to be too big to enter the pores of both 0.515 and 0.6 nm. As shown in Fig. 2b, c, CH_4_ molecules are found to be restricted to the gas box without molecule distribution in the channel or offside vacuum box. In the gas box, a pronounced peak of adsorbed CH_4_ is found near the membrane, which is attributed to the strong electrostatic interaction between CH_4_ and membrane. All these indicate that the membrane with pore size of 0.515~0.6 nm can be as a molecular sieve, where small H_2_ molecules can pass preferentially, whereas large CH_4_ is forbidden to penetrate.

**Table 1:** Number of gas molecules entering the channel of GM and corresponding CH_4_ selectivity

Parameters	0.515 (nm)	0.6 (nm)	0.64 (nm)	0.728 (nm)	0.855 (nm)	1.111 (nm)	1.366 (nm)
H_2_	55	61	74	77	80	82	86
CH_4_	0	0	86	145	129	121	110
*S*(CH_4_/H_2_)	0	0	1.20	1.88	1.61	1.47	1.28

#### W = 0.64 − 1.366 nm

At the pore width of 0.64 nm, CH_4_ molecules start to enter the channel. At 10 ns, 86 CH_4_ molecules have diffused into the pore (Table 1). For H_2_, the amount of entered molecules increases to 74 at 0.64 nm. The corresponding selectivity of CH_4_ over H_2_ is found to be 1.39. At the pore width of 0.728 nm, the amounts of entered CH_4_ and H_2_ molecules increase to 145 and 77, respectively. The CH_4_/H_2_ selectivity also increases to 1.88 at 0.728 nm. However, when the pore size further increases to 0.855, 1.111, and 1.366 nm, the amount of entered CH_4_ molecules gradually decreases to 129, 121, and 110, respectively, although more and more H_2_ molecules (80, 82, and 86) diffuse into the channel. Correspondingly, the CH_4_/H_2_ selectivity decreases to 1.61, 1.47, and 1.28 for the pores of 0.855, 1.111, and 1.366 nm, respectively. Therefore, it can be seen that with the increasing pore width from 0.515 nm to 1.366 nm, the number of H_2_ molecules entering the pore increases gradually, whereas a pronounced peak of CH_4_ is found at 7.28 Å with the maximum CH_4_/H_2_ selectivity of 1.89.

Similar to the pores of 0.515 and 0.6 nm, the concentration profiles suggest that most of H_2_ molecules, which go into the pore, could penetrate the membrane to the offside vacuum boxes at 0.64 − 1.366 nm (Fig. 2d-h). However, CH_4_ molecules entering the pore prefer to stay in the channel with few molecules passing into the offside vacuum box, especially the channel of 0.728 nm, indicating an optimal CH_4_-adsorbed pore of 0.728 nm. As a lightweight nonpolar molecule, the main interaction of H_2_ with graphene sheets is the weak Van der Waals terms. Therefore, H_2_ molecules could easily pass through the membrane without accumulation in the channel, and the H_2_ permeability is proportional to the width of the channel (active surface area). For CH_4_ molecules, the strong electrostatic interaction with graphene sheets results in the packing of methane in the channel.

**Fig. 3:**
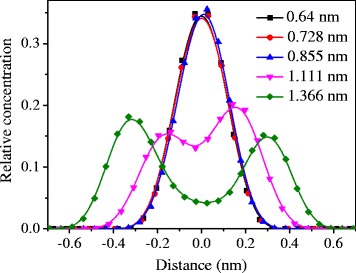
Concentration profile of the final configurations for CH_4_ molecules along the *x* direction at the middle of the channel as a function of channel width

**Fig. 4:**
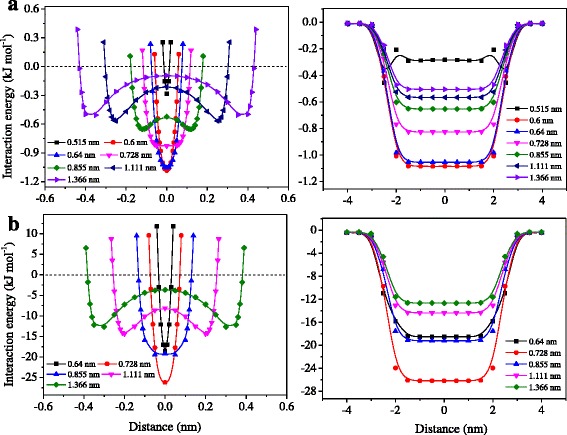
Interaction energy of **a** H_2_ and **b** CH_4_ molecules with GM as a function of channel width along the (*left*) *x* and (*right*) *z* directions

To further explore the transport mechanism of CH_4_ in the channels, the density profiles of CH_4_ across the channel (*x* axis) as a function of pore width were plotted (Fig. 3). As shown in Fig. 3, at the pores of 0.64, 0.728, and 0.855 nm, a striking peak of CH_4_ is located at the center of the channels, suggesting that CH_4_ molecules diffuse in the middle region of the channel via a single layer. When the pore is bigger than 1.1 nm (1.11 and 1.366 nm), the transport of CH_4_ is found to be near the two surfaces of channel via the double molecular layers, as reflected by two strong peaks of CH_4_ density profiles. A similar situation was also reported by Vieira-Linhares and Seaton using the DCV-GCMD method [19], where the methane molecules at graphite pore of 0.7 nm are most effectively packed in a single layer, while a double-layer adsorption of methane is found at 2.0 nm. Figure 4b shows the profile of interaction energy between CH_4_ and graphene surface across the channel (i.e., along *x* axis). This indicates that a single potential energy well at the center of the pore is located for the channels of 0.64, 0.728, and 0.855 nm, with the deepest well of 0.728 nm (26.2 kJ mol^−1^), while a double potential energy well is formed in the channels of 1.11 and 1.366 nm and is found to be shallower than that in the 0.64~0.855 nm channels. These explain that the adsorption of CH_4_ is via the single and double layers in the channels of 0.64~0.855 and 1.11~1.366 nm, respectively, with the optimum adsorption condition of 0.728 nm. In addition, among the pore sizes of 0.64~1.366 nm, 0.728 nm also bears the largest interaction energy with CH_4_ along the whole *z* axis of channels (Fig. 4b), which further reflects the maximum CH_4_ adsorption capacity on the pore of 0.728 nm.

For H_2_ molecules, the channels show a similar single and double potential energy well at the width of 0.515~0.64 and 0.728~1.366 nm, respectively (see Fig. 4a). However, all the energy wells are very shallow, which are less than 1.09 kJ mol^−1^. Therefore, H_2_ molecules are unfavorable to adsorb on the channel surfaces, and the main factor influencing H_2_ permeability is the active surface area (pore width) rather than the interaction energy.

### Edge Functionalization

Five functional groups were considered to explore the effect of edge groups on the transport properties of the channel, i.e., hydrogen (–H), fluorine (–F) hydroxyl (–OH), amine (–NH_2_), and carboxyl (–COOH). The pore widths of 0.6, 0.64, 0.728, and 1.336 nm are selected as models of sieving membrane, monolayer adsorbed membrane, optimum membrane, and bilayer adsorbed membrane, respectively.

#### W = 0.6 nm

At the pore width of 0.6 nm, we can find the number of H_2_ molecules entering the channel follows the order GM–F (89) > GM–OH (81) > GM–H (75) > GM (61) > GM–NH_3_ (47) > GM–COOH (0) (see Table 2). This order may be attributed to the different polarities and sizes of the functional groups. Compared to the pristine edge of graphene, the polar –F, –OH, and –H groups show a stronger intermolecular interaction (Van der Waals terms) with H_2_ molecules, which could improve the diffusion of H_2_ into the channel. For the –NH_3_ and –COOH ligands, they have much larger volume, resulting in a smaller active surface area at the entrance of channel. Therefore, compared to the pristine GM, the permeability of H_2_ is found to decline for GM–NH_3_ and even decreases to zero for GM–COOH.

**Table 2:** Number of gas molecules entering the channel of edge-functionalized GM and corresponding CH_4_ selectivity

Widths (nm)	Parameters	GM	GM–H	GM–F	GM–OH	GM–NH_2_	GM–COOH
0.6	H_2_	61	75	89	81	47	0
	CH_4_	0	0	0	0	0	0
0.64	H_2_	74	79	85	87	69	0
	CH_4_	86	90	105	77	0	0
	*S*(CH_4_/H_2_)	1.20	1.14	1.23	0.88	0	0
0.728	H_2_	77	85	82	82	81	11
	CH_4_	145	145	145	142	8	0
	*S*(CH_4_/H_2_)	1.88	1.71	1.76	1.73	0.10	0
1.366	H_2_	86	88	91	90	92	85
	CH_4_	110	112	109	109	111	107
	*S*(CH_4_/H_2_)	1.28	1.27	1.20	1.21	1.21	1.26

**Fig. 5:**
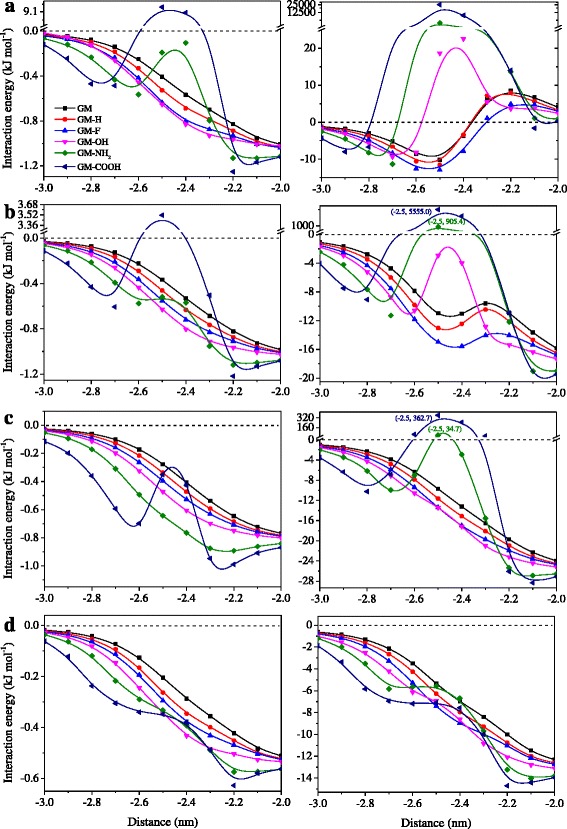
Interaction energy of (*left*) H_2_ and (*right*) CH_4_ molecules with pristine and edge-functionalized GMs along the *z* direction near the entrances with the width of **a** 0.6, **b** 0.64, **c** 0.728, and **d** 1.366 nm

To further understand the effect of edge functionalization on H_2_ permeability, we calculated the interaction energy between gas molecules and the surface of channel along the *z* axis. It is found that the edge groups have negligible influence on the interaction with H_2_ molecules at the center of channel but show a large effect near the entrance of channel (near −2.5 nm along the *z* axis, see Fig. 5). The edge functionalization by –F, –OH, and –H groups could effectively strengthen the interaction energy with H_2_ at the entrance of channel, with the sequence of GM–OH > GM–F > GM–H > GM. For GM–NH_2_ and GM–COOH, however, they form an energy barrier at the entrance, especially GM–COOH ligand, where the energy barrier is much higher than the energetic zero (the energetic sum of free H_2_ molecule and GM). All these suggest the promotion of H_2_ transport for –F, –OH, and –H groups but blocking H_2_ penetration for –NH_3_, especially –COOH. Note that the contrary order of interaction energy to H_2_ permeability for GM–F and GM–OH may be attributed to a smaller active surface area of GM–OH than that of GM–F at the entrance, which will be discussed in the following.

#### W = 0.64 nm

At the pore size of 0.64 nm, both H_2_ and CH_4_ molecules could enter the channel of pristine GM. When the edge is functionalized, the amount of H_2_ molecules entering the pore is enhanced for GM–H (79), GM–F (85), and GM–OH (87), whereas –NH_3_ and –COOH groups (69 and 0) play a negative role, similar to the situation at 0.6 nm. For the CH_4_ permeability, only –H and –F groups (90 and 105) play a positive role, while a negative influence is found for –OH, –NH_2_, and –COOH groups, especially –NH_2_ and –COOH, which still completely reject CH_4_ molecules to enter the channel. Therefore, we can find that after edge functionalization, the CH_4_/H_2_ separation selectivity for GM–H (1.14) and GM–F (1.23) is comparable with that for GM (1.20), but an inverse CH_4_/H_2_ selectivity (0.88) is presented for GM–OH. Furthermore, when the edges of GM are functionalized by the bigger –NH_2_ and –COOH groups, GM–NH_2_ becomes an H_2_ molecular sieve, while GM–COOH remains to completely prohibit the penetration of both H_2_ and CH_4_ molecules.

The profiles of interaction energy at 0.64 nm pore indicate that similar to the situation at 0.6 nm, interaction energy with H_2_ molecules at channel entrance is strengthened after edge functionalization by –F, –OH, and –H groups, while an energy barrier is formed at the entrance of –NH_2_ and –COOH functionalized membranes but lower than that at 0.6 nm (Fig. 5b). For CH_4_ molecules, it is interesting that an energy well is formed at the entrance of GM–F, GM–H, and GM (Fig. 5b), which may be caused by the strong electrostatic interaction between CH_4_ molecules and the polar edge of the membranes. Furthermore, the order of depth of energy well (GM–F > GM–H > GM) is in accordance with that of CH_4_ permeability. However, because of a steric effect, GM–OH, GM–NH_2_, and GM–COOH form a high energy barrier at the entrance to restrain the pass of CH_4_ molecules. Especially, the extra high energy barrier of GM–NH_2_ and GM–COOH results in a zero probability of CH_4_ penetration at 0.64 nm.

**Fig. 6:**
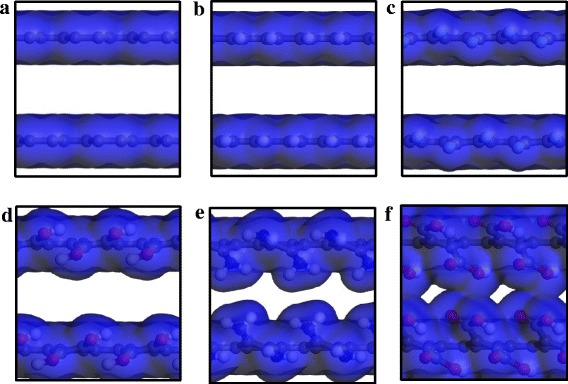
Electron density isosurface of the entrances of **a** GM, (**b**) GM–H, (**c**) GM–F, (**d**) GM–OH, (**e**) GM–NH_2_, and (**f**) GM–COOH (isovalue of 20 e nm^−3^)

The permeability of gas molecules is also associated with active surface area of the channel entrance. Therefore, we calculated the electron density isosurface of the entrance for the pristine and edge-functionalized GMs at 6.4 nm (see Fig. 6). As shown in Fig. 6, both –F and –H groups almost have no influence on the pore shape of the entrance, suggesting a comparable active surface area of GM–F and GM–H with pristine GM. For GM–OH, its active surface area is slightly less than that of GM, GM–H, and GM–F. Therefore, –OH group has an inhibiting effect on the permeability of big CH_4_ molecules, while the influence on small H_2_ molecules is negligible for the pore of 0.64 nm but relatively large for the small pore of 0.6 nm, as discussed above. After edge functionalization by NH_2_, the active surface area of GM–NH_2_ at the entrance becomes apparently small and thus has a negative effect on the H_2_ permeability and is even too small to allow CH_4_ molecules to enter. For the biggest –COOH group, it forms an absolutely leak-tight screen to prevent the penetration of both H_2_ and CH_4_ molecules.

#### W = 0.728 nm

When the pore width increases to 0.728 nm, all of the pristine and functionalized GMs (–F, –H, –OH, –NH_2_, and –COOH) allow H_2_ molecules to pass. Compared to pristine GM, the amount of H_2_ molecules entering the channel increases to 81~85 for GM–H, GM–F, GM–OH, and GM–NH_2_, except GM–COOH, where only 11 H_2_ molecules could enter. For CH_4_ molecules, permeability of GM–F, GM–H, and GM–OH is up to maximum (142~145) at 0.728 nm, similar to GM (145). However, only eight CH_4_ molecules enter the pore of GM–NH_2_, even there is still no CH_4_ molecule diffusing into the pore of GM–COOH. As shown in Table 2, the CH_4_/H_2_ selectivity for GM–H, GM–F, and GM–OH (1.71 ~ 1.73) is slightly less than that for GM (1.88), while a much low value (0.10) is found for GM–NH_2_, indicating an inversely high H_2_ selectivity. In addition, GM–COOH can be as a molecular sieve at pore size of 0.728 nm to allow only H_2_ to pass.

The interaction energy between gas molecules and the membrane was also calculated at pore size of 0.728 nm (see Fig. 5c). As shown in Fig. 5c, the H_2_ energy barrier at the entrance of GM–NH_2_ disappears, according with the comparable permeability of H_2_ with GM, GM–H, GM–F, and GM–OH. In addition, the height of H_2_ energy barrier for GM–COOH has become lower than the energetic zero, suggesting the acceptability of H_2_ transport. For CH_4_ molecules, both GM–NH_2_ and GM–COOH form an energy barrier at the entrance of the channel. However, the barrier for GM–NH_2_ is relatively low and could be overcome by methane molecules, whereas the barrier for GM–COOH is still too high to be overcome.

#### W = 1.366 nm

At the large pore size of 1.366 nm, the values of H_2_ and CH_4_ permeability for both pristine and functionalized membranes fluctuate in a small region of 85~92 and 107~112, respectively, suggesting the influence of edge functionalization is slight for the large pore. As discussed above (see Fig. 4), for both H_2_ and CH_4_ molecules, the interaction energy profiles (i.e., along *x* axis) at 1.366 nm show a double potential well across the channel with location near the surfaces of membranes. Although edge functionalization has some effects on the potential well at the channel entrance (Fig. 5d), the large active surface area may play an important role in gas transport at big 1.336 nm pore and thus results in a comparable gas permeability for all the considered membranes.

## Conclusions

The separation of binary H_2_/CH_4_ mixture through the 2D graphene channels has been investigated via molecular simulation calculations. The results show that for the pristine GM, the membrane with a pore width of 0.515~0.6 nm can be a molecular sieve, which allows small H_2_ molecules to enter but forbids large CH_4_ to pass. Although both H_2_ and CH_4_ molecules could transport into the 0.64~1.366 nm channels, a favorable selectivity of methane over hydrogen is observed, with a maximum value of 1.89 at 0.728 nm.

The edge functionalization of GM could modify the active surface area of the pore and tune attractive and/or repulsive interaction with molecules at the entrance of channel, which has a striking influence on the transport of H_2_/CH_4_ mixture. (1) At the small pore width of 0.6 nm, the edge modification by –H, −F, and –OH groups could improve the H_2_ permeability of molecular sieve, but a negative effect is observed for –NH_3_, especially –COOH, which completely prohibits hydrogen to penetrate. (2) A preferential CH_4_/H_2_ selectivity is found for GM–H and GM–F at pore widths of both 0.64 and 0.728 nm, respectively, while the selectivity for GM–OH changes form H_2_ molecules at 0.64 nm to CH_4_ molecules at 0.728 nm. For GM–NH_2_, it always favors transport of H_2_ at pores between 0.64~0.728 nm, as reflected by an excellent hydrogen molecular sieve property at 0.64 nm and a significant H_2_/CH_4_ selectivity at 0.728 nm. With the increasing channel of GM–COOH up to 0.728 nm, gas molecules begin to enter the pore but are restricted to small H_2_ molecules. When the pore width further increases to 1.336 nm, the influence of edge functionalization becomes weak, resulting in a comparable CH_4_/H_2_ selectivity for all the considered membranes.

## Additional File

Additional file 1:
**Supporting information.** **Fig. S1.** Final configurations of the 1:1 H_2_/CH_4_ mixture permeating through the 2D channel of pristine and edge-functionalized GMs (DOCX 4515 kb)
