# Supplementary material for: Insights into the H2/CH4 Separation Through Two-Dimensional Graphene Channels: Influence of Edge Functionalization
Source: Nanoscale Res Lett. 2015 Dec 23;10:492. doi: 10.1186/s11671-015-1199-2 (PMC4689719; doi:10.1186/s11671-015-1199-2)
Supplement: Additional file 1: — Supporting information. Fig. S1. Final configurations of the 1:1 H2/CH4 mixture permeating through the 2D channel of pristine and edge-functionalized GMs (DOCX 4515 kb) [file 11671_2015_1199_MOESM1_ESM.docx]

**Supporting Information**

**Insights into the H_2_/CH_4_ separation through two-dimensional graphene channels: Influence of edge-functionalization**

Jing Xu,^1^ Pengpeng Sang,^1^ Wei Xing,^1^ Zemin Shi,^1^ Lianming Zhao,^1*^ Wenyue Guo,^1^ Zifeng Yan^2*^

*^1^ College of Science, China University of Petroleum, Qingdao, Shandong 266580, PR China*

*^2^ State Key Laboratory of Heavy Oil Processing, Key Laboratory of Catalysis, China University of Petroleum, Qingdao 266580, PR China*

*Correspondence should be addressed to lmzhao@upc.edu.cn (L. Z.);* [*zfyancat@upc.edu.cn*](mailto:zfyancat@upc.edu.cn) *(Z. Y.)*

**Fig. S1** Final configurations of the 1:1 H_2_/CH_4_ mixture permeating through the 2D channel of pristine and edge-functionalized GMs.
